# Hospital-visiting pregnant women signal an increased spread of hepatitis C infection in Khyber Pakhtunkhwa region of Pakistan

**DOI:** 10.1186/s12985-017-0861-y

**Published:** 2017-10-10

**Authors:** Zobia Afsheen, Bashir Ahmad, Shumaila Bashir

**Affiliations:** 1 Department of Microbiology and Biotechnology, Abasyn University Peshawar, Peshawar, Khyber Pakhtunkhwa Pakistan; 2 Centre for Biotechnology and Microbiology, University of Peshawar, Peshawar, Khyber Pakhtunkhwa Pakistan; 3 Department of Pharmacy, University of Peshawar, Peshawar, Khyber Pakhtunkhwa Pakistan

**Keywords:** Elisa, Hepatitis C virus, Immuno chromatographic technique, PCR, Pregnancy

## Abstract

**Background:**

Seroprevalence of hepatitis C in Khyber Pakhtunkhwa province of Pakistan was determined by screening blood samples of expectant mothers seeking antenatal care in gynecological units of district hospitals. The rationale behind this cohort study was that the availability of free-of-cost antenatal care in district hospitals brings expectant mothers from a broader geographical range in each district and thus provides a large sample-size of healthy pregnant women of known medical history for Hepatitis C Virus (HCV) surveillance. The study was carried out along a south west to north east transact of five districts, Kohat-Peshawar-Nowshera-Charsadda-Mardan, with the central district Peshawar and outer districts Kohat and Mardan bordering northern mountainous ranges of the Khyber Pakhtunkhwa province. This distribution of districts along the transact allowed the study to gauge the impact of proximity to remote highland communities on the HCV burden of visiting pregnant women tested for HCV infection.

**Methods:**

The cohort study randomly selected 150 pregnant women visiting each hospital for serological screening for Anti-HCV carried out by ELISA assay. The feasibility of ICT and RT-PCR assays for HCV prevalence was also examined in the present study.

**Results:**

With a total of 750 blood specimen screened, the results of ELISA tests revealed a staggering 5.9% frequency of Anti-HCV in the five districts with the frequency of ELISA positive cases ranging from 3.3% in Nowshera, 4.7% in Charsadda, 6.0% in Peshawar, 6.7% in Kohat, and 8.7% in Mardan. The relatively higher frequencies of Anti-HCV cases among hospital visiting pregnant women in Peshawar, Kohat and Mardan were consistent with the proximity of these hospitals to the highland communities in the bordering mountain ranges. Compared to 44 Anti-HCV positive serologic specimens detected by ELISA, only 26 and 10 blood specimens were tested positive by ICT and PCR methods, respectively. Our study validates ELISA as a reliable diagnostic technique for both acute and chronic HCV infection.

**Conclusion:**

The HCV infection rate of 5.9% in Khyber Pakhtunkhwa province clearly exceeds the HCV prevalence rates reported for other regions in Pakistan, making this province a hotspot of HCV infection in the country.

## Background

Hepatitis C viral infection is a major health issue globally. It is a liver disease for which no vaccine is available at present; however, it is a treatable disease with about a 95% cure rate [1]. Following the onset of an infection, HCV disease in its acute phase can cause jaundice and can lead to serious health complications in expectant mothers [2–5]. During pregnancy, viral hepatitis is found to be associated with a high rate of vertical transmission, high risk of maternal complications, neonatal and fetal hepatitis. It has also been reported as a major cause of maternal mortality [6–9].

The progression of hepatitis C to its chronic phase occurs in about 55–85% of infected cases [10] and untreated patients face an increased risk of developing liver cancer, liver cirrhosis or both [11]. According to a recent global estimate [12], 185 million people around the world are living with HCV disease, which corresponds to a global prevalence rate of about 2.8%. However, there are marked differences in the regional prevalence of HCV around the world. In Eurasia, the Eastern Mediterranean region with a HCV prevalence rate of 2.3% is characterized as a major endemic zone in the world [10]. On the continental scale HCV prevalence is considered to be highest in Africa [13, 14]. In the South East Asian region, Pakistan has been identified with one of the highest burdens of HCV in the world; however, the estimates of the HCV prevalence in Pakistan have varied between 0.3-31% [15–18]. HCV infection in Pakistan is on the rise: in a recent investigation carried out by the Centers for Disease Control and Prevention (CDC) [19], many clusters of new HCV infections were identified and the territories within the province of Khyber Pakhtunkhwa were characterized as one of the most active regions reporting new cases of HCV infection. The aim of the present study was to delineate the spread of HCV infection in Khyber Pakhtunkhwa.

## Methods

### Study area

The study was hospital-based and sampling was carried out at district headquarter hospitals of Kohat-Peshawar- Charsadda- Nowshera- and Mardan located along a south-west to north-east transact starting from the Kohat mountain range running through Peshawar valley and ending at the foothill of the Malakand mountain range (Plate1). Proper ethical clearance was obtained before commencing the study in these district hospitals.

### Study population

Seven hundred and fifty (750) blood samples (150 samples from each hospital) were obtained from women attending antenatal clinics at district headquarter hospitals. Informed consent was taken from every participant before blood collection.

### Collection and processing of blood samples

Venipuncture was performed and after disinfection with 70% alcohol, 5 mL blood from the arm was collected in a vacutainer for hepatitis C serological tests. After 8–10 min at room temperature, the blood sample was centrifuged at 7155 *g* for 5 min and the serum was transferred to three separate tubes for diagnostic procedures. ICT strip test was performed onsite and the collected sera were transported on ice to the lab and stored at −20 °C for ELISA and PCR diagnostic procedures.

### Immuno-chromatographic screening for HCV antibodies

The onsite screening for anti-HCV antibodies was carried out by immunochromatographic assay (ICT) using Standard Diagnostics BioLine HCV (Multi) Strips. The validity of each strip result was confirmed by the appearance of a positive test line accompanied by a control test line.

#### ELISA test for HCV antibodies

All 750 serologic specimens were tested for Anti-HCV Antibodies using a microplate ELISA assay system (Biocell Anti-HCV E0320). The assay was carried out as described in the Biocell kit, and the ratio (R) of sample absorbance to the calculated cut-off absorbance was used to identify HCV positive samples as recommended by the manufacturer.

#### Detection of HCV-RNA

Real Time Polymerase Chain Reaction (RT-PCR) was carried out using a Sacace HCV Real-TM Qual kit for the qualitative detection of HCV in serologic specimens using the following procedure:

HCV RNA was extracted from sera following the manufacturer’s instructions (Sacace, REF K–2-C/100). RT-PCR was performed for each sample using a Cepheid Smart Cycler system and the detection was carried out using two reporter dyes monitored at two different wavelengths, one for HCV and the other for Internal Control (IC). Fluorescent intensities during RT-PCR were monitored to determine accumulated product and the cycle threshold (Ct) value, i.e. the number of PCR cycles required to exceed the IC fluorescence signal, was determined for each sample.

Samples yielding a Ct value lesser than 40 were considered positive for HCV-RNA, while those yielding a Ct value greater than 40 were considered PCR negative.

## Results

**Fig. 1:**
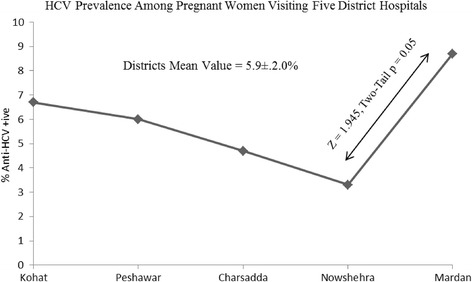
HCV frequency in pregnant women visiting five district hospitals in Khyber Pakhtunkhwa.150 pregnant women were screened at each district hospital

**Fig. 2:**
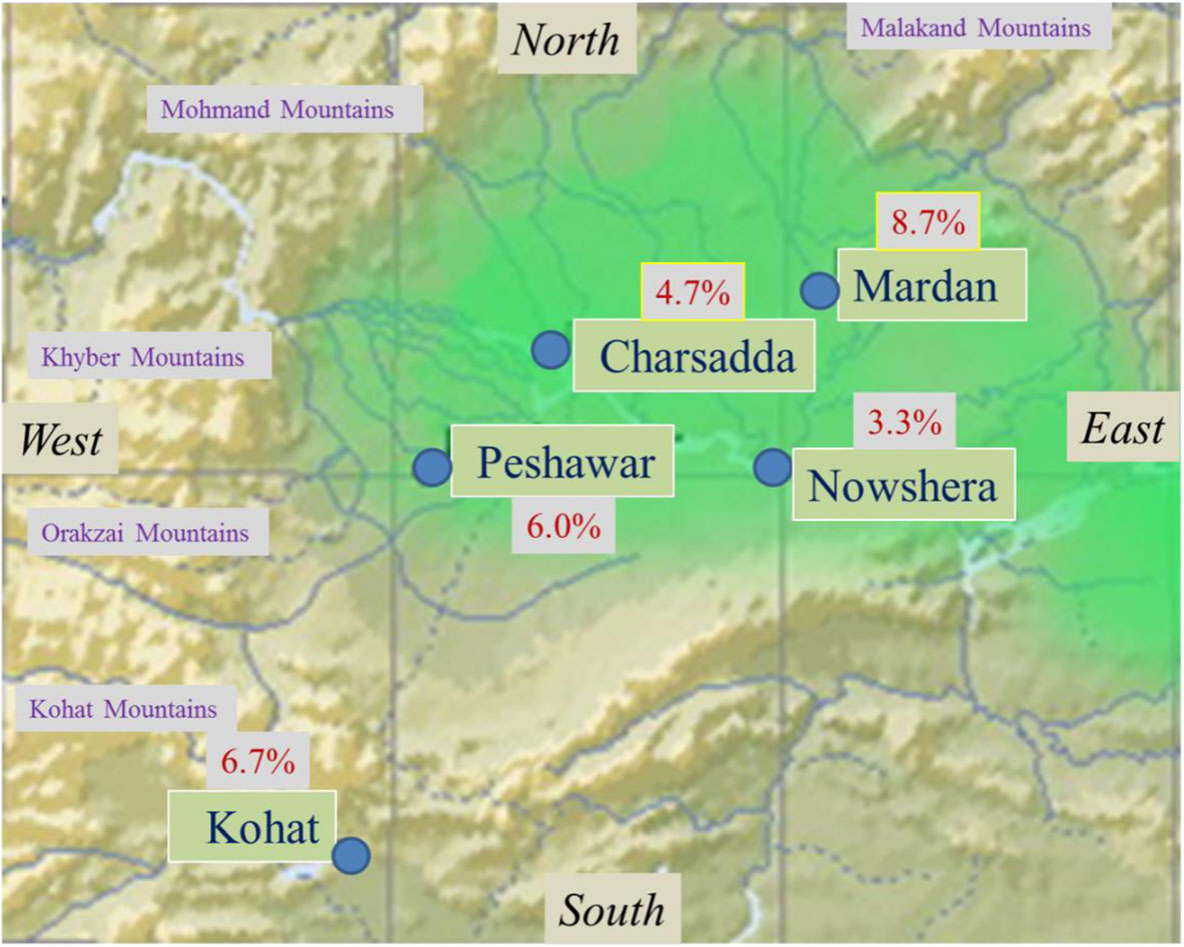
Map of five districts screened in Khyber Pakhtunkhwa, Pakistan. The prevalence levels are shown as percentages. The green shaded area encompasses Peshawar valley

Of the 750 ELISA diagnostic procedures carried out on serologic specimens from hospital- visiting pregnant women in five district hospitals, 44 tested positive for the presence of anti-HCV antibodies, corresponding to a HCV prevalence rate 5.9 ± 2% in the area (Table 1). The prevalence rate showed a considerable variation at the district level. Peshawar, the largest central district along the south-west to north-east transact, was characterized by an intermediate frequency of 6% positive cases, while the two other central districts, Nowshera and Charsadda, showed a lower frequency of 3.3% and 4.7%, respectively. The two outer districts, Kohat and Mardan, showed a higher frequency of 6.7% and 8.7%, respectively (Table 1, Figs. 1, 2). The sharpest difference in the HCV positive frequency was observed between the two adjacent districts Nowshera and Mardan, which was found to be significant (Fig. 1) by a two-tailed probability test (z = 1.945, *p* = 0.05).

**Table 1:** ELISA anti-HCV positive blood samples from pregnant women visiting five district hospitals in Khyber Pakhtunkhwa

	Kohat	Peshawar	Charsadda	Nowshera	Mardan	Districts Total
Samples Tested	150	150	150	150	150	750
Anti-HCV + ive	10	9	7	5	13	44
% Anti-HCV + ive	6.7%	6.0%	4.7%	3.3%	8.7%	5.9%

The numbers of HCV positive cases detected by ICT and RT-PCR procedures were 26 and 10, respectively (Table 2). These values correspond to 41% and 23%, respectively, of serologic specimens identified as HCV positive by the ELISA procedure (Tables 1, 2). ELISA was therefore significantly more sensitive than ICT (*X* ^2^ = 20.5, *p* > 0.005) and RT-PCR (*X* ^2^ = 29.9, p > 0.005) in detecting HCV infection in serologic specimens.

**Table 2:** ICT Anti-HCV and RT-PCR HCV-RNA positivity among pregnant women visiting five district hospitals in Khyber Pakhtunkhwa

District	ICT	RT-PCR
Kohat	3	1
Peshawar	5	3
Charsadda	2	1
Nowshera	10	2
Mardan	6	3
Total	26	10

## Discussion

Hospital visiting pregnant women in Khyber Pakhtunkhwa exhibit a staggering HCV infection rate of 5.9%, which is higher than the 4.7% estimated for Pakistan [16] and more than double the global average of 2.3% [10]. Pakistan has been categorized with one of highest burdens of HCV in the world, exceeding the HCV prevalence rate of its neighboring countries [16]. The present study, by revealing a marked variation in the HCV prevalence among pregnant women visiting gynecological units in different hospitals, appears to endorse the existence of high HCV prevalence pockets in the northern regions of Pakistan. Of the five hospitals surveyed in the present study, only two central districts, Nowshera and Charsadda, were within the 3–5% range of HCV prevalence reported for the country. In comparison, the three other district hospitals - Peshawar, Kohat, and Mardan - were much higher in their frequency of HCV infection, with Mardan almost doubling the nationwide estimate of 4.7% [15].

The only plausible factor linked to a two-fold variation in the frequency of HCV positive cases among pregnant women visiting five district hospitals appears to be the geographical position of the hospital. The two hospitals with the lower HCV positive cases are clustered within the center of Peshawar valley. District Peshawar is also situated within the plains of Peshawar valley, nonetheless it borders on the west and north with a long belt of Orakzai, Khyber and Mohmand mountains. These mountain ranges are sparsely populated with few healthcare facilities available to remote highland communities, and accessing hospital facilities in Peshawar is the common route of obtaining healthcare in these remote areas. Similarly, the district hospital in Kohat is accessible to highland communities living in Orakzai, Kurram and Waziri hills, and the district hospital in Mardan reporting the highest prevalence of 8.7% is accessible to the vast Malakand mountain range in the north. It is worth noting that even with the expected overlapping along the five districts transact surveyed here, the difference between Mardan and its adjacent Nowshera district was found to be statistically significant in two-tailed probability test. Khyber Pakhtunkhwa is clearly a high HCV prevalent province in Pakistan, and its regional variations indicate the presence of rapidly HCV spreading pockets in several districts. A larger multi-centered study of each district in Khyber Pakhtunkhwa would be needed to further delineate the regional pattern of HCV in this high HCV prevalence province.

Recently, Jiwani and Gull [20] have issued a stern warning regarding the uncontrolled rise in HCV prevalence in remote areas of Pakistan; the authors link this rise in infection to poverty, poor health care facilities and a lack of awareness in these neglected regions. There have been reports of high HCV prevalence in the remote mountain communities of Khyber Pakhtunkhwa [21, 22]. It can be argued therefore that an influx of expectant mothers from potentially high HCV prevalent remote communities seeking antenatal hospital facilities in Peshawar, Kohat, Mardan and Kohat may have added to the HCV burden in these gynecological units.

The increased burden of HCV infection in expectant mothers in high prevalent areas is a serious risk for both mothers and their newborn infants, as it can lead to life-threatening complications if not diagnosed early and left untreated. There is therefore an urgent need for systematic surveillance of HCV infection in Khyber Pakhtunkhwa. The surveillance needs to take into account the specific concerns about the uncontrolled HCV spread in rural and remote highland communities of northern Pakistan [20].

Over the past 20 years, the estimates of HCV infection in Pakistan have been marred by a problematic range of 0.3 to 31% prevalence discrepancy from published reports [15, 16]. One of the major limitations in HCV surveillance in Pakistan has been the lack of standardization for the detection of viral infection. Recently, CDC has established an ELISA based viral hepatitis surveillance system for Pakistan [19]. ELISA is the most feasible procedure available for HCV screening at this point in time. The present study has found ELISA by far the more sensitive than ICT and RT-PCR procedures. Both ELISA and ICT are Anti-HCV antibody assays and are designed to detect HCV presence in serologic samples, however, ICT in the present study gave negative results for nearly half of the samples identified as HCV positive in ELISA tests. ICT has been found to produce both false negatives and false positives in serological HCV studies [23]. The main attraction of the ICT strip test is its low cost and technical ease. However, its reliability as a diagnostic tool is unacceptably limited. The RT-PCR procedure detects the HCV RNA in serologic specimens and its usefulness is limited to the detection of chronic phase of infection. RT-PCR results are usually negative during the acute phase of infection. The present study, therefore, recommends adopting the CDC system of ELISA based viral surveillance [19] for delineating the spread of hepatitis C in rural remote communities in Khyber Pakhtunkhwa. A timely surveillance of HCV infection is important to control the disease as the newly available drugs Ledipasvir and Sofosbuvir have been highly effective in eradicating the virus in HCV patients and achieving an over 95% cure rate [24].

## Conclusion

The present study screened pregnant women visiting five district hospitals. The HCV infection rate of 5.9% in Khyber Pakhtunkhwa province clearly exceeds the HCV prevalence rates reported for other regions in Pakistan, making this province a hotspot of HCV infection in the country. At the district level, the study found a strong link between the geographical location of the hospital and the frequency of HCV infection among pregnant women visiting the hospital. The levels of infection in the two central districts of Nowshera and Charsadda were lower and closer to the national average than in the three mountain bordering districts Peshawar, Kohat and Mardan. Remote highland communities in Pakistan have been reported to be suffering an uncontrolled spread of HCV, and the higher burden of HCV in pregnant women visiting Kohat, Peshawar, and Mardan hospitals appears to provide a circumstantial evidence of HCV proliferation among the highland communities of Khyber Pakhtunkhwa. These results indicate an urgent need for province-wide surveillance of HCV spread in Khyber Pakhtunkhwa.

## Abbreviations

CDC: Centers for Disease Control and Prevention
Ct: Cycle Thresh-hold
ELISA: Enzyme Linked Immuno-Sorbent Assay
HCV: Hepatitis C Virus
IC: Internal Control
ICT: Immuno Chromatographic technique
RT-PCR: Reverse Transcriptase Polymerase Chain Reaction
